# The changing landscape of professional practice in podiatry, lessons to be learned from other professions about the barriers to change – a narrative review

**DOI:** 10.1186/s13047-019-0333-2

**Published:** 2019-04-16

**Authors:** Michael Harrison-Blount, Christopher Nester, Anita Williams

**Affiliations:** University of Salford, Salford, UK

**Keywords:** Barriers, Change, Organisations, Professional awareness, Service users

## Abstract

**Background:**

The delivery of healthcare is changing and aligned with this, the podiatry profession continues to change with evidence informed practice and extending roles. As change is now a constant, this gives clinicians the opportunity to take ownership to drive that change forward. In some cases, practitioners and their teams have done so, where others have been reluctant to embrace change. It is not clear to what extent good practice is being shared, whether interventions to bring about change have been successful, or what barriers exist that have prevented change from occurring. The aim of this article is to explore the barriers to changing professional practice and what lessons podiatry can learn from other health care professions.

**Main body:**

A literature search was carried out which informed a narrative review of the findings. Eligible papers had to (1) examine the barriers to change strategies, (2) explore knowledge, attitudes and roles during change interventions, (3) explore how the patients/service users contribute to the change process (4) include studies from predominantly primary care in developed countries.

Ninety-two papers were included in the final review. Four papers included change interventions involving podiatrists. The barriers influencing change were synthesised into three themes (1) the organisational context, (2) the awareness, knowledge and attitudes of the professional, (3) the patient as a service user and consumer.

**Conclusions:**

Minimal evidence exists about the barriers to changing professional practice in podiatry. However, there is substantial literature on barriers and implementation strategies aimed at changing professional practices in other health professions. Change in practice is often resisted at an organisational, professional or service user level. The limited literature about change in podiatry, a rapidly changing healthcare workforce and the wide range of contexts that podiatrists work, highlights the need to improve the ways in which podiatrists can share successful attempts to change practice.

## Background

Healthcare in the UK has rapidly changed in response to the changing epidemiology of diseases, the impact of chronic and debilitating conditions, complicated by an aging population [1–4]. This is adding to increased demands on the National Health Service (NHS) and the cost of care [1, 5]. Ham [6] identified that transformation is required in how we deliver care to make improvements in efficiency. Successive governments have highlighted the need for the healthcare workforce to develop, innovate, adapt and transform themselves and services to meet these demands, and this includes the podiatry workforce [1–4, 7–9].

Podiatry practice continues to evolve, where the expansion in the scope of practice and successful legislative closure have been important strategic steps [10–12]. In service terms, the focus is the clinician led development of sustainable, evidence based foot care services, that provide a patient focused, high-quality, safe, accessible, bio psychosocial model of care [1].

‘High-need, high-cost’ patients with multiple or complex conditions are heavy users of all aspects of care [13]. A quarter of the population will be over 65 by 2033 [4], and as people age they are likely to have more than one long-term health condition along with increasing frailty [14]. Demand for podiatry services from these patients is high, because complex diseases with multiple co morbidities and polypharmacy put the lower limbs at increased risk of injury and subsequent disability.

The challenge of meeting these demands for podiatrists is complex and requires on-going change of working practices, role flexibility, skills and underpinning knowledge [15, 16]. ‘Allied Health Professions into Action’ identifies the Allied Health Professionals (AHPs) transformative potential to lead change, with the benefits of improving health, care and wellbeing of individuals and populations [2].

At the same time podiatrists are tasked with putting patient care at the centre of all they do [3, 8]. Indeed, the clinical case for changing professional practice is evidence that people who are more informed, involved, able and confident to self-manage their conditions, generally experience better health and quality of life than those who are not [17–19]. The facilitation of shared responsibility is especially important for those with long-term conditions and thus the priority groups for podiatry services [11, 12]. Podiatry practice has to change to support this, but we have no clear evidence at this stage in its evolution, that this is happening.

Changing established clinical practice and professional behaviors is difficult and results are often not readily embraced and therefore short lived [8, 20–24]. Healthcare professionals need to be supportive of autonomy and feel empowered to change behaviors [22]. Forced change is often short lived and elicits reactance [21]. A variable response to the need for change leads to ever more variable clinical practices and likely varied experiences and outcomes for patients.

Furthermore, many ‘change’ projects fail, and the most commonly cited reason is neglect of the human dimensions of change, [25, 26]. This neglect often centres on a lack of insight into why people are unhappy with organisational change, a poor appreciation of the process of change, and a limited knowledge of the tools and techniques that are available to bring about change. Resistance to change can be considered as normal, and can be useful to reveal issues that need to be addressed and resolved, before successful implementation of new methods. It is often the perception and process of the change that is resisted, rather than the change itself [27–29].

A key part of understanding the success and failure of change depends on how far people within an organisation understand and deal with the change process [30]. Lewin’s (1951) cornerstone model for understanding organisational change sets out a framework for managing change known as Unfreeze – Change – Refreeze. Recognising these three distinct stages facilitates the planning to implementation phases of the change process. This framework is transitional and for transition between phases to occur it needs to be free from barriers. The unfreeze stage concerns the motivation, understanding, attitudes and perceptions of change, making this phase crucial to beginning the change process.

Therefore, in order to effectively explore change it is important to first identify potential factors that prevent clinicians changing their practice, and the lessons learned [31]. Research into changing professional practice has tended to focus on the nursing and medical professions [28, 29, 31–39], and very few studies have investigated change in podiatry practice. In responding to the need for change at professional, service and individual clinician levels, it is important that members of the podiatry profession seek to learn from other professions that have also faced change. It is important to understand what factors will influence a podiatrist’s ability to identify the barriers they may encounter.

The aim of this article was to explore the barriers to changing professional practice in healthcare and whether the lessons learnt could be applied to the profession and practice of podiatry.

## Methods

A literature search was carried out which informed a narrative review of the findings. The inclusion criteria were publications in the English language literature, using the search terms “behaviour change”, “podiatry”, “professional practice”, “barriers to change”, “professional boundaries” and “interventions” from 2008 to March 2018. Searches were carried out using Scopus, MEDLINE, PubMed, Springerlink, CINAHL, The Cochrane Library, Wiley online library, Oxford University press, British Nursing Index, SAGE journals, SCIverse, directory of open access journals (DOAJ). Reference lists of identified articles; the indexes of journals from which articles were retrieved and key reviews were also searched. An initial search revealed a paucity of literature related to changing professional podiatry practice. The search criteria were therefore widened to include other health care professions. A number of systematic reviews have stated the difficulty in searching this area are due to its broad nature, the literature being in both generalist and specialist publications and being poorly indexed in bibliographical databases [24, 40].

Papers considered eligible for inclusion had to (1) examine the barriers to change strategies in a healthcare setting, (2) explore knowledge, attitudes and roles during change interventions, (3) explore how the patients/service users contribute to the change process (4) include studies from predominantly primary care in developed countries.

Quantitative studies were included if they were primary research investigating the barriers and facilitators to changing professional practice published in English. Qualitative studies that focused on broader questions were included if changing professional practice and/or service user contribution to influence change was a clear focus of at least one aspect of analysis.

## Results

A total of 92 articles were included in the final narrative review with the results organised into themes. This included 13 nursing texts, 19 medical texts (medicine and primary care) 6 dental texts, 6 from allied health professional research and 48-health policy, social care and related texts.

Thirteen initial barriers were derived from the literature. These initial barriers were then organised into 3 overarching themes for discussing change (Table 1).

**Table 1:** Barriers to change identified in the literature

Barriers to change	Themes to discuss barriers to change
• Poor practice organisation (time constraints/ environment/ clinical setting/ finances). • Support (Failure to provide access to appropriate information or time for innovation and training). • Failure of previous change initiatives.	The Organisation (The existing structures, operations, culture and context of the change environment)
• Individual practice, habits, tradition and personal preferences. • Knowledge (Unaware of current evidence based practice (EBP), obsolete clinical knowledge/ skills/ inappropriate professional development (CPD)). • Aware guidance has changed but unaware of how to adopt change or how to access new evidence. • Fear (clinical uncertainty/ loss of professional identity/ autonomy/ inability to perform as well in the new situation. • Inadequate training, communication and preparation for the change effort. • Lack of incentives to participate in effective educational activities or change processes.	The Professional (Awareness, knowledge and attitudes)
• Demands for care. • Media influence. • Perceptions and beliefs about appropriate care. • Compliance with clinical guidance.	3 – The patient (As a consumer and a co-producer of care)

## Discussion

The literature will be discussed under the three overarching themes identified; the organisation (existing structures, operations, culture and context of the change environment), the professional (awareness, knowledge and attitudes), the patient (as a consumer and a co-producer of care).

## The organisation

Podiatry operates in very varied organisational contexts with little formal organisation around early career mentorship and the development of specialist roles, and so we should expect change to be a variable activity for the profession and the individual [41, 42]. Podiatry practitioners may work within complex healthcare organisations, individually, as part of a multi-disciplinary clinical team or working in the private sector as business owners or within a private health care organisation. Therefore, different organisational structures, cultures and associated clinical settings will form part of the environment that research has shown can facilitate or hinder change [8, 25, 43, 44].

The nature of the organisation will impact on the chances of change occurring [45–47]. Indeed, sustaining change is dependent on an organisations ability to adapt to either an external or internal need for change, [8, 45–47]. Failure rates with change across a wide range of organisations including healthcare are high, with 60–70% of change initiatives failing [43–47]. These failures have been attributed to organisational culture, driven by negative workforce attitudes and unproductive management behaviour. The drive for healthcare organisations to change comes from the need to improve quality and meet financial constraints [1, 3, 4]. It is underpinned by business cases that focus on the movement toward patient centered care [1–4, 7–9]. ‘AHPs into action’ (2017), states that a commitment is required to ensure that evidence based treatments and interventions are delivered with the preferences and values of the person, given the same consideration as the clinician’s expertise in the decision making process. This commitment is consistent with The Health Foundation’s description of person centred care [48].

This, however, relies on the ability to refocus organisational policy and care delivery around the patient, supported by evidence for improving clinical outcomes and patient experiences [49]. This process is complicated by the fact that the change is being implemented in an environment of mixed professional identities littered with both financial and practical constraints. Previous research into the role of the health care setting has shown that it is key that an organisation wide approach is needed to bring about change, and attention is payed to the key barriers that may limit success. These include inadequate resourcing of the redesign of care delivery, insufficient resources, failures building staff capacity, not ensuring accountability, failing to focus on the needs of the service user and a culture unsupportive of change and learning needs [33, 36].

Organisational time pressures have been identified as a barrier to both organisational and personal change with competing and more immediate demands taking precedent over preparation for change and patient satisfaction, [50–55]. Wallis [54] highlighted that the two most frequently cited barriers to change are lack of time and an unsupportive organisational culture. In an Australian study, 149 nurses indicated that 2 of the10 main barriers to changing practice were insufficient time on the job to implement new ideas and not enough time to read research [55]. Change is needed if we are to increase patient satisfaction and quality of care [1, 3, 4, 7–9, 43–47]. Change may be continuous, sporadic, occasional, or rare. Predictable change allows time for preparation, whereas unpredictable change is more difficult to respond to effectively [56].

To achieve patient satisfaction, healthcare organisations require staff to spend more time with patients. Several US based studies demonstrate that time spent educating a patient can have an important bearing on patient satisfaction. Patients who felt they had not spent enough time with a practitioner were less satisfied [57–59].

Dugdale et al. [60] explored the effects of limiting time on the doctor-patient relationship and concluded that a visit rate of greater than 3 or 4 patients/hour, may lead to “suboptimal visit content”. Freeman et al. [61], reviewed outcomes of the debate about length of consultation and reported longer consultations were associated with a range of better patient outcomes, particularly with mental health*,* and recommended that longer consultations should be a priority. Wilson and Childs [62] published a systematic literature review of 13 papers in which they explored associations between consultation length and consultation process and process and healthcare outcomes. They concluded that the overall evidence suggests that a patient who spends more time with their doctor is more likely to have a consultation that includes important elements of care [60–63].

However, this time is not infinite and has to be stolen from elsewhere. Increasing the time spent with patients to build better quality patient practitioner relationships may impede effective planning and preparation for organisational or personal change. Those in the private sector may spend more time with patients as part of a more customer rather than patient focused approach [60]. To protect time with each patient more hours may be worked. Furthermore private practitioners may use time outside of scheduled work hours to explore the research and identify possible new revenue streams to enhance practice. Whereas in a public sector setting, practitioners may choose to work more or faster during scheduled hours, since the organisation may set constraints on the start and end of days with little flexibility, or protected time for other activities. Other studies have demonstrated that it is not the actual time spent with the healthcare professional that affects outcome, but rather what happens during that time [64, 65]. Those patients of healthcare professionals with a “participatory decision-making style”, improved patient–practitioner communication patterns and greater expression of emotions, overall had better health outcomes, were more satisfied and were 30% less likely to seek alternative care [47, 57, 58, 66, 67]. Since changes in healthcare occur so rapidly, they are less likely to be predictable [68]. Therefore, the capacity needs to exist within the system to adopt change effectively.

Practitioners in private healthcare practice have reported the difficulties of higher levels of accountability, the demands of running a business and the requirement to maintain standards of proficiency, as barriers to finding time to change their clinical practice [69]. Since 2002 the Health and Care Professions Council have adopted an outcomes led development model that replaced the previous model of CPD point accumulation, to demonstrate improvement and change in practice. This change highlights the need for practitioners to develop an approach of lifelong learning. This includes implementing research, demonstrating professional development through reflective practices from a number of educational activities, or engagement in experiential learning opportunities, which are more than just an accumulation of points. Time constraints have been identified as a barrier to the behavioural change required to adopt this style of learning [50–53]. This learning style can be perceived by practitioners to take too much time, due to researching evidence, appraisal, literature searching and reflecting on experiences. Private practitioners can bill a patient for the time spent directly providing care or indirect activities relating to ongoing care such as orthotic manufacture. It is not common practice however for the practitioner or practice to bill a patient for the time taken to attend a course, complete a personal development portfolio or research the evidence to support a treatment plan.

Professional colleagues and networks are important to support change [1–3]. Research into occupational therapy practices has shown that private sector working can lead to isolation, heightened accountability, administrative demands, and concerns over time and money [69]. Working in a community setting and in private practice presents an inherent isolation, which has described practitioners as ‘islands’ [69]. Sole occupational therapy and dental practitioners have been faced with the challenge in taking the initiative to make contacts with fellow professionals, at a frequency that allows for discussion about specific issues, which may ultimately influence a change in practice [32, 69]. Involvement in formal and informal professional networks improves collaboration and has a major impact on change [70, 71]. Practitioners, who did not belong to any network and were professionally isolated, lacked these opportunities [32, 69]. Furthermore, it is not clear from the reviewed literature how collaboration is encouraged [70–72]. When working in a group setting (i.e. hospital, or health centre) information gets shared spontaneously and motivates the practitioner to inquire more about ways to improve and change practice [32, 69]. This contrasts with evidence that dental practitioners have to actively seek out support when faced with clinical uncertainties [1, 2, 67, 72]. A review highlighted positive results from interprofessional learning in areas such as patient satisfaction, clinical error and clinical competencies [73]. However, due to the limited number of studies and heterogeneity of the change interventions, conclusions had limited generalisability [73].

Most reviews on behaviour change highlight the importance of the organisational level and how to bring about change [74]. However, there is little research into interventions targeting organisational change, and processes that have failed or not gained momentum. This has been attributed to the possible difficulties establishing a comparable control group, as organisational healthcare contexts will all differ [75]. A recent survey of Charities trying to instigate change, found that 50% of change processes instigated haven’t worked [76]. They concluded that several factors relating to the organisational structure and leadership had led to these failures. These included, the change process going on too long, running out of steam and not being followed through, commitment was poor, the change was poorly communicated and a failure to identify how the change would benefit the organisation. This meant that results were short lived, old behaviours and attitudes crept back in, and systems did not change to support the initiatives [76].

Previous research into the role of the health care setting has shown that it is key that an organisation wide approach is taken to bring about change and attention is payed to the key facilitators that will ensure success. These include adequate resourcing of the redesign of care delivery, sufficient resources, actively building staff capacity, improving the way in which practitioners work, ensuring accountability, a focus on the needs of the service user and a culture supportive of change and learning needs [61, 62, 77]. Communicating the vision of the organisation, creating and guiding coalition and getting the right people in charge, is key to making the ideas stick and gaining traction for the process to succeed. Organisations that have been through change processes and succeeded in fostering patient-centered care have gone beyond mainstream frameworks for quality improvement based on clinical measurement and audit, and have adopted a strategic organisational approach to patient focus where patients and practitioners have changed their behaviour to influence and bring about change in the organisational context.

## The awareness, knowledge and attitudes of the professional

Working in multiprofessional settings convey many benefits to both the patients and the health professionals [78, 79]. These include improved health outcomes and enhanced satisfaction for patients, and the more efficient use of resources and enhanced job satisfaction for team members [77–79]. This overall better mindset and the desire and connections to drive bottom-up change can have a positive outcome for healthcare delivery. The potential benefits of multidisciplinary working and building networks across health care teams and organisations has been highlighted in articles and policy documents in recent years [77–80]. These documents suggest that large-scale change is only possible when professionals work jointly [81–83]. It has been shown that effective change to complex systems such as hospital services requires team effort and close group working, [84]. Furthermore, the complexity of a patients needs will require all professionals to adopt multidisciplinary approaches to service delivery, regardless of an acute or community setting, [85]. Carmel, highlighted how healthcare professionals in ‘close knit’ ICU teams have obscured the perceived doctor-nurse professional boundary with an informal working environment, close working relationships and a sensitivity to one another’s viewpoints, [86]. This has however had the effect of reinforcing organisational boundaries where joint allegiances to the unit are far stronger than those to colleagues from different departments, [86]. A change to effective cross sector working requires prerequisites to collaboration, such as physical proximity, social proximity, positive motivation, a strategy of incorporation and the size of the team’s membership. All of which will promote decision-making, communication and the effect of increasing the influence of all of the professional groups involved, [86, 87].

However, changes to professional identity and the blurring of roles challenges a professional’s beliefs, values and behaviours, introducing uncertainty and instability during periods of change, [88]. Podiatrists work in co-existence with other professional groups and differences in professional culture can lead to controlling behaviours, affect performance and the response to adopt change, [86, 88]. Loss of professional autonomy and erosion of professionalism, employment status differences, and cultural differences between professions, geographical separation and membership of multiple teams have been highlighted as barriers to change in multidisciplinary teams, [87]. A qualitative analysis of two collaborative nursing schemes in England showed that professional identity and roles changed in response to cross sector working, leading to role ambiguity, role erosion and in some cases role extension [89]. Researchers exploring cross sector working with GPs and social services identified both professions wanting the other to change its culture of working and concerns were raised about power and hierarchy [90, 91]. Furthermore, a lack of recognition from management, leads to staff feeling undervalued, disengaged and less likely to be flexible to taking on new approaches to working [13]. The nursing literature [13, 14] has highlighted issues around the change process being complicated, medical and other professional groups being unsupportive or unaware of new research that was underpinning the case for change, the false sense of hierarchy from some research nurses and the preferences of doctors taking precedence over new practices [14].

Specialisation, professional advancement and changes in skill sets are all seen as positive changes to advance a professional group, however professional pioneering can also act as a barrier to change because it leads to some practitioners being left behind. Nancarrow et al. stated that professional roles remain safe whilst there continues to be a demand for their specialised skills [16]. However, to improve outcomes for patients and ‘work smarter’, professionals must also be willing to diversify and adopt change to retain professional boundaries, secure new roles and take ownership of the technologies used to provide them [92]. Boundaries of practice are therefore not fixed quantities. The emergence of health care assistant (HCAs) roles has been found to blur role boundaries and threaten professional identity, with perhaps inevitable unwillingness to change having implications for teamwork, quality of patient care, and patient safety [93]. Research with podiatrists suggests that innovations such as use of podiatry assistants is welcomed and important to reduce the profession**’** s wider vulnerability to boundary encroachment by other professions and roles [94]. It is thought that podiatry assistants could provide a greater proportion of core foot care, and it has been suggested that more nurses could carry out low risk foot assessments in people with diabetes [95, 96]. Furthermore, a delegation of tasks to podiatry assistants increases accessibility to foot-care services, reduces waiting times for low risk procedures and increases clinical availability of more specialised podiatrists, [94]. Willingness to change and adopt a model of care where assistants take on greater roles in other professions has been met with more skepticism. The medical profession is concerned with the number of physician assistants that will be employed, perhaps at the expense of doctors [97]. Furthermore, nurses’ report feeling deprived of their “hands on” role as HCAs rather than they, interact more and build relationships with patients [98, 99]. Skepticism and therefore resistance to embrace health care assistant’s roles as a positive change exists in the private sector, with some believing podiatry assistants could leave assistant practice and set up as non-Health and Care Professions Council (HCPC) registered foot health practitioners and therefore be a threat to their businesses.

Advances in technology, the spectrum and complexity and the environment of less independence are all having an effect on the “final product” of the generalist podiatrist. This along with changing healthcare economics and the public demand of specialisation mean practitioners must adapt. Progressive specialisation [100], has evolved in other previously generalist healthcare services due to the complex environment of change in both medical education and the healthcare system. Podiatry practice however does not, apart from in podiatric surgery have a postgraduate foundation to provide a focused, intensive educational experience in a recognised subspecialty area. Specialisation within the podiatry profession as with others needs to be managed carefully to ensure work continues to be safe, pertinent and within the scope of professional practice knowledge [100, 101]. Those not willing to change and support greater specialisation risk isolation within the healthcare system, and those who do change need to be cautious that this does not lead to a dilution of expert and the loss of the professional ‘all-rounder’ [100–102]. Either extreme in these scenarios results in people working across broad areas and not willing to change, or within niche specialisations, working according to their own individual agendas and having little clarity regarding who should be taking on which tasks [100, 101, 103].

The importance of evidence to support clinical decisions is well established and the evidence base for podiatry and foot health care is growing. The role of evidence-based practice (EBP) divides the health professions with its supporters and detractors. Overall allied health professionals strongly support EBP and the importance of consulting the evidence base [104]. For some however EBP is seen as only one tool in a range of approaches to improving patient care and, at its worst, as an impediment to providing bespoke holistic care [105]. Whilst clinical judgment remains indispensable and ensures the practitioner is the adjudicator over available evidence, studies in the United States and Europe suggest that 30–50% of patients do not receive care according to current scientific evidence [70, 106]. With the rapid development of new interventions or assessment techniques, it is unclear what initiates a change in practice and thereafter improvements in patient outcomes. Good clinical research is inherently slow to conduct and publish, with an average of 17 years between completing the research and it been implemented in a set of guidelines [107]. Research included the podiatry profession cite professional attitude, lack of time, skills, funding and opportunities to contribute to creation or use of evidence as important barriers to changes in practice [108–110]. Many practitioners have reported that both the evidence base and their own ability to access and understand it were inadequate [104, 105]. Some believe the literature can be too obscure and irrelevant to everyday practice [111], and thus do not search or read literature that could inform changes in practice.

Employers (and contractors of health services) are responsible for ensuring their workforce is competent and able to meet the needs of the service. This should include development of skills, access to the resources (including the time to learn) and supportive planning. Organisations that have a culture of learning and change alongside information systems that allow staff to measure their outcomes and the quality of care they provide, help to better maintain and improve performance [110]. Increasing clinician’s knowledge and skills in the use and implementation of evidence-based practice into their daily work can be achieved through development and exposure to educational interventions and activities. Keeping clinicians informed and motivated this way has been shown to bring about change in clinical practice [24, 25]. The role of students within health care practice has been seen as one solution to provide this daily exposure [69]. This partnering of skills has been shown to be effective in helping clinicians to develop research skills and for the student to acquire ideas of the types of clinical relevant questions that need to be researched to inform and improve practice. Subsequent involvement in change/improvement projects has also shown to be an effective way of changing clinician behaviors [25, 69]. It was also recognised that this is more difficult in a private practice situation where there is often less access to students.

Clarification around the uncertainty about professional roles and professional accountability, the fear of undermining autonomy, lack of familiarity of latest EBP, and/or the lack of skills to know how to integrate it into clinical practice have all been identified as internal barriers (i.e. those values pertaining to the practitioner) to change in health care practice. It is important to recognise that as professionals our internal values facilitate change. It is clear from the literature that one of the most important of these is the fear around the implementation of EBP specifically the lack of skills and abilities to search and critically appraise the literature accurately [69, 111–113]. In a competitive work environment or private practice setting there is an inherent resistance to share intellectual property with colleagues and/ or competitors. Overcoming the barriers to using EBP will be eased if practitioners are willing to adopt the belief that finding, sharing and using the evidence is important to minimise time, account for lack of skills and improve patient benefit [69].

## The patient as a consumer

Patient factors are a powerful influence over change. Patients are being encouraged to be more influential in the development of health policy and in the care that they receive, both of which drive change. Patients with access to greater amounts of information about their health needs are perceived as having either positive and/ or negative influences on the treatment decisions and practice of clinicians [114, 115]. Changes in society and technology means that patients have greater access to information about their health, and consequently have higher expectations of the type and quality of care they will want to receive, where they will receive it and how long they will have to wait [116]. Additionally, patients are increasingly taking a more decisive role in their own treatment and may no longer be simply content to take the first treatment offered [32]. Examples from the dental literature show that discussions with patients and patient values are two of the main factors governing a clinician’s treatment philosophies and willingness to change [32, 67].

Contemporary patients appear to be better educated, more assertive and skeptical of health care providers [117]. Open access to the Internet medical databases allows patients to use the same sources of information as healthcare practitioners, but often without the critical skills to appraise the evidence and confounding this is the absence of quality control of the information available [45, 118, 119]. As a result, some patients may not trust the practitioner’s knowledge or judgment [117]. Consequently, clinicians are forced to find more radical ways to change practices as simply reading journals, attending continuing professional development (CPD) courses, and consulting colleagues, may not be enough to satisfy some patients’ demands for assurance of treatment effectiveness. Treatments that are offered are often those that can best be described as the practitioner’s routine, or that which the practitioner has determined is the therapy the patient would most likely choose [119]. Patients, however, frequently assume that the practitioner is offering the best or the only treatment available for their condition [67].

Patient’s wishes and preferences have been seen to directly influence treatment decisions, for example a request for a certain type of treatment or intervention despite it being unnecessary or inappropriate from an evidence basis. In one study 71% of physicians stated that patients’ wishes had influenced their decision to admit patients to an intensive care unit [120]. Similarly, general practitioners in Iceland were found to be influenced by patients’ pressure to prescribe antibiotics in unnecessary cases [121]. Patients’ treatment preferences have been found to influence the type of management decisions in 7% of dermatology outpatient consultations [122]. For example, patients may prefer creams over ointments because ointments are sticky. In this regard by giving patients what they want rather than what is required patient satisfaction in the short term may be achieved and they are more likely to be satisfied and compliant with treatment regimes. In a private setting this may convert to loyalty to the practice, and positive service user feedback within NHS organisations. Where the patient is well informed and challenges outdated practices this can bring about positive change. However, patient preferences may go against the medically optimal treatment option, and this false positive has a negative effect, as it provides no impetus for practitioners or organisations to change their practices.

Patients can be influential in bringing about radical change in how we deliver twenty-first century healthcare, but this requires the relationship between the health sectors, health professional and patient to change to make it happen [114]. The current healthcare system continues to have tightly defined boundaries between professional and recipient, limited by a professional-knows-best mindset. This lack of acknowledgement of factors that shape health outside of the boundaries of the healthcare system, restrict professionals in seeing the possibilities for patients to be active in bringing about change [123]. NHS England have highlighted the need for professionals and health services to have a new relationship with patients and communities where collectively healthcare initiatives will help shift power to patients and citizens, [1]. These active patients or ‘patient leaders’ are identified as those patients, users and carers who have the confidence and capability to influence change. Their main purpose is to improve health and well-being in the community and/or improve health and social care services by working cumulatively with others to influence decision- making [124]. This ‘Co-production’ means delivering public services where people’s needs are better met because they are involved in an equal and reciprocal relationship with professionals and others, working together to get things done, [125]. Where activities are co-produced in this way both services and factors outside of the health care system are equally recognised and together become far more effective agents of change, [125–128]. Service users and their families have different expectations about how their relationships with services, and professionals, should be [129]. Orientating services around principles of recovery and personalisation involves recasting relationships between service users and professionals as true partnerships.

In private practices with a stable patient base, where trust and rapport have been established, implementing change was more likely [129]. It could be said that an advantage exists here for practitioners and patients when personalised care is provided, because as well as being an essential aspect of good care; continuity has also been shown to bring about change to improve safety, efficiency and effectiveness of the services offered [129].

In current NHS primary and secondary care where services may be stretched, locations of services changed, and staff under pressure to deliver care in a set time, personalised and continuity of care cannot always be achieved. Current guidelines for diabetes care for example highlight the need to adopt an individual approach that takes into account personal preferences, comorbidities, polypharmacy risk and a patient’s ability to benefit from long-term interventions [87]. This influence can be a strong positive driver for change, however the time taken to provide individual health care, acknowledging a patient’s individuality and the unique way, in which they experience a condition, may have cost as well as time implications. It has been argued however, that the benefits of safety and effectiveness would offset the extra cost and time spent providing this type of care [129]. Changes in practice to provide individual care and improve continuity also supports the importance placed upon developing good communication skills and trust to enable practitioners to fully explain treatment options and enhance the patients experience, [129].

The role of the patient to drive change in health care systems is not the norm, but the value of co-production spans the entirety of our healthcare system. On a small scale, factors that in some circumstances may be perceived as barriers to uptake can also act as levers for change. For example, patients may influence practitioners’ behaviour towards clinically effective practice by requesting interventions of proven effectiveness. Opinion leaders may also influence practitioners positively, and the media may promote cost-effective interventions. The bigger picture explores the change roles patients have to play in shaping future health care.

## Summary

The limitations of the narrative review are that there is a lack of podiatry-centered research. There remain several unanswered questions. We currently have no idea whether podiatrists change their practice, or if they do what it is that they are changing. Nor do we know which area of practice is subject to the most change and why this is so. Further, we do not know what factors are most influential in determining a change or what mechanisms are used to implement change. This review has focused on studies from predominantly primary care in developed countries. With this approach we still need to exercise some caution when drawing comparisons between practices. Although we may all share similar drivers for change, such as ageing populations and the need for greater chronic illness management. We do not necessarily share the same organisational infrastructures and legislative processes within our systems to make this transfer of knowledge an equitable one. These organisational differences have a profound impact on what professions from differing nations can or cannot change.

Lewin’s change model includes both drivers of and barriers to change [30]. The driving forces are to push the organisation into a state or direction in which the change can occur. Lewin stated that restraining forces (barriers) could hinder change and push the organisation in the opposite direction. Despite the fact that Lewin mentioned restraining forces during the change process, less emphasis is put on identifying them. His focus remained on the change process itself. However without first addressing these forces the change process cannot begin.

To begin any successful change process, you must first start by understanding why the change must take place. Lewin [30] stated that, “Motivation for change must be generated before change can occur. One must be helped to re-examine many cherished assumptions about oneself and one’s relations to others.” This is the unfreezing stage from which change begins [30]. To change successfully therefore clinicians are forced to, challenge their beliefs, values, attitudes, and behaviors that currently define their practice. When Lewin’s framework is applied to the podiatry profession evidence is in short supply at all 3 stages of his change process. However, there is substantial literature on implementation strategies that overcome barriers to changing professional practice in other health professions. Hence, this review has revealed information about the barriers to change in professional practice and the strategies that can be adopted in order to support effective change within the podiatry profession. This has resulted in this review being a UK centric discussion around change.

## Conclusion

Most knowledge of barriers to and incentives for change are not derived from well-designed prospective studies, but rather from observational studies and theoretical reflections. The evidence around difficulties in implementing change has led most experts in healthcare improvement to now emphasise that in order to be most successful, change initiatives must have a compelling vision and provide explicit guidance to organisations, teams and individuals about how to change, rather than just what needs to change.

Effective change has been seen through adoption of a strategic approach to a learning organisation culture that supports and facilitates change with a patient focus. Studies have highlighted that adoption of a learning organisation culture can, stimulate new ideas, develops teamwork, facilitates effective change processes and promotes quality improvement in healthcare. Podiatry needs to explore more participative, multi-faceted and interactive educational approaches that appear to have a greater long-term impact on producing sustained changes in clinical practice.

To deliver bottom-up change, flexibility is required within the system both in terms of the formal organisational structure and day to day processes. Multidisciplinary working and collaboration between peers within and between organisations lead to broader operational and personal benefits. Operationally and personally change programmes can be more effective, collaborative working builds more supportive working relationships, which is essential to improve health outcomes for people and populations. A move towards more multidisciplinary working practices for podiatry could therefore nurture a more positive mind-set and the desire to change.

The influential role of patient’s/ service users in the change process, or implementation strategies at the levels of the organisation and wider context is also underexplored in the literature. The podiatry profession should take a variety of steps to ensure that patients and carers are able to act as agents of change as a norm in our practice. This goes beyond traditional patient involvement, to co-production and patient leadership, which requires a paradigm shift in how podiatry services approach patient and carer engagement. This approach relies on professionals who are prepared to listen, share power and to move out of the expert role into a partnership role, [130, 131].

The wide and diverse range of barriers that limit practitioners’ ability to change their routine clinical practices need to be addressed. Identifying the domains of podiatric practice necessary for competent practice may provide a framework for ensuring that key skill areas of podiatry can be targeted by change initiatives. A future vision for podiatry would be to encourage an open learning culture in which podiatrist’s share successful and unsuccessful attempts to change practice. We have an opportunity to make this cultural shift by teaching and embedding the skills for adaptation and change early on in the undergraduate experiences so that the acceptance of change becomes the podiatrist’s cultural ‘norm’.
